# Milled surgical guides vs. freehand placement of orthodontic implants: a preclinical micro-CT study

**DOI:** 10.3389/froh.2026.1834583

**Published:** 2026-06-18

**Authors:** Alexander Schwärzler, Danijel Domic, Sophia Panwinkler, Patrick Chitan, Benedikt Sagl, Erwin Jonke

**Affiliations:** 1 Clinical Division of Orthodontics, University Clinic of Dentistry, Medical University of Vienna, Vienna, Austria; 2 Clinical Division of Oral Surgery, University Clinic of Dentistry, Medical University of Vienna, Vienna, Austria; 3 Etiology and Therapy of Periodontal and Periimplant Diseases (ETEP) Research Group, Faculty of Dentistry, Complutense University of Madrid, Madrid, Spain; 4 Competence Center Artificial Intelligence in Dentistry, University Clinic of Dentistry, Medical University of Vienna, Vienna, Austria

**Keywords:** CAD/CAM milling, digital orthodontics, guided implants, miniscrews, orthodontic implants, skeletal anchorage, TAD

## Abstract

**Objective:**

The present study evaluated the transfer accuracy of computer-aided designed and manufactured (CAD/CAM) milled guides compared with freehand placement of orthodontic temporary anchorage devices (TADs).

**Methods:**

Sixty TADs were placed in 30 typodonts, using either CAD/CAM-milled guides or conventional freehand insertion. The freehand insertions were performed by one orthodontist and one oral surgeon. Micro-CT scans were obtained to determine actual TAD positions. Deviations from planned positions were assessed via vector analysis in vertical, horizontal, and transversal axes, as well as angular displacement. Linear mixed models with Items as random effects were applied to evaluate the influence of group and quadrant as fixed effects while accounting for repeated measures.

**Results:**

Guided placement demonstrated significantly higher accuracy across almost all linear and angular dimensions compared with freehand insertion (*p* < 0.01). Greater accuracy was shown for the oral surgeon compared with the orthodontist for freehand placement (*p* < 0.01). Inter- and intra-observer agreement was excellent.

**Conclusion:**

CAD/CAM milled guides significantly improved TAD placement accuracy compared with freehand insertion. This preclinical study supports their potential clinical benefit in single-session guided workflows, particularly for enhancing precision across different operator experience levels.

## Introduction

Orthodontic mini-screws and temporary anchorage devices (TADs) have substantially improved orthodontic therapy by increasing the predictability of anchorage control, an essential component of orthodontic therapy (1). Recently, a “one-visit protocol” was described by De Gabriele et al. (2017) in which TAD insertion with transfer guide and placement of a prefabricated Computer-aided Design and Manufacturing (CAD/CAM) appliance are completed in a single appointment, thereby enhancing significantly clinical efficiency (2, 3). Guided techniques allow not only preoperative virtual planning and placement of TADs, but they also enable the simultaneous design of associated orthodontic appliances, such as expanders, distalizers, or intrusion devices (4, 5), thus improving overall treatment precision while reducing uncertainty. However, to successfully perform a one-visit protocol, a highly accurate TAD placement is essential (6).

To support a similar one-visit workflow in another field of dentistry, oral implantology, a computer-assisted implant surgery (CAIS) was developed (7). Static surgical templates represent powerful tool for prostheticaly driven implant placement and can enable same-day prosthetic delivery, while minimizing the risk of injury to adjacent anatomical structures (7, 8). Notably, the transfer accuracy of digitally planned implant placement has been extensively investigated, with numerous clinical studies demonstrating superior accuracy compared with freehand implant placement (9). For example, a recent systematic review and meta-analysis reported a mean difference of 2.11 (0.94–3.28) degree of angular deviation, and mean differences in linear deviations of 3.89 mm (1.61–9.38) at the implant head and 1.39 mm (0.03–2.76) at the tip of the implant (9). In addition, Shusterman et al. (2024) similarly reported that static guided systems reduced angular and linear deviations at both the implant head and apex compared with freehand methods (10).

In general, surgical guides can be manufactured using either subtractive techniques (milling) or additive manufacturing methods (3D-printing) (11). While 3D printing has gained increasing popularity, milled guides are often regarded as more accurate (12–14). Despite the growing clinical adoption of 3D-printed guides due to their lower cost and faster in-office production, the comparative accuracy of milled guides for orthodontic TAD placement remains insufficiently investigated, warranting further evaluation (15). To the authors’ knowledge, while previous studies have compared freehand TAD placement with 3D-printed guide-assisted insertion, no studies have directly compared CAD/CAM-milled guides with conventional freehand TAD placement in orthodontics. Although TAD insertion may be performed by orthodontists or referred to oral surgeons, no study has specifically compared placement accuracy between these two specialists (16).

This study aimed to address these gaps by evaluating whether subtractive manufacturing (milling) improves the precision of TAD placement compared with freehand techniques. In addition, the study explored whether freehand placement accuracy differs between an orthodontist and an oral surgeon. The objective was to compare linear and angular deviations following freehand versus milled-guide TAD insertion. The null hypothesis assumed no significant differences in transfer accuracy between freehand insertion and placement using CAD/CAM-milled surgical guides.

## Materials and methods

**Figure 1:**
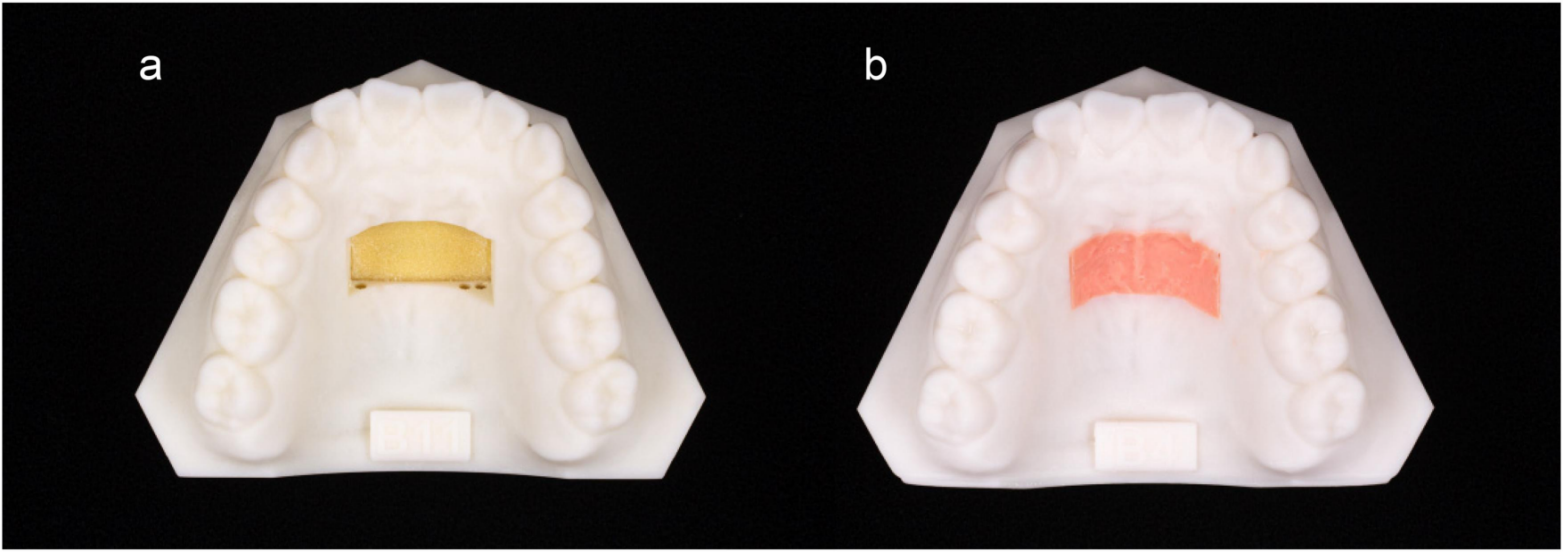
Typodont including an artificial bone **(a)** which was covered with a gingiva mask to simulate gums **(b)**.

**Figure 2:**
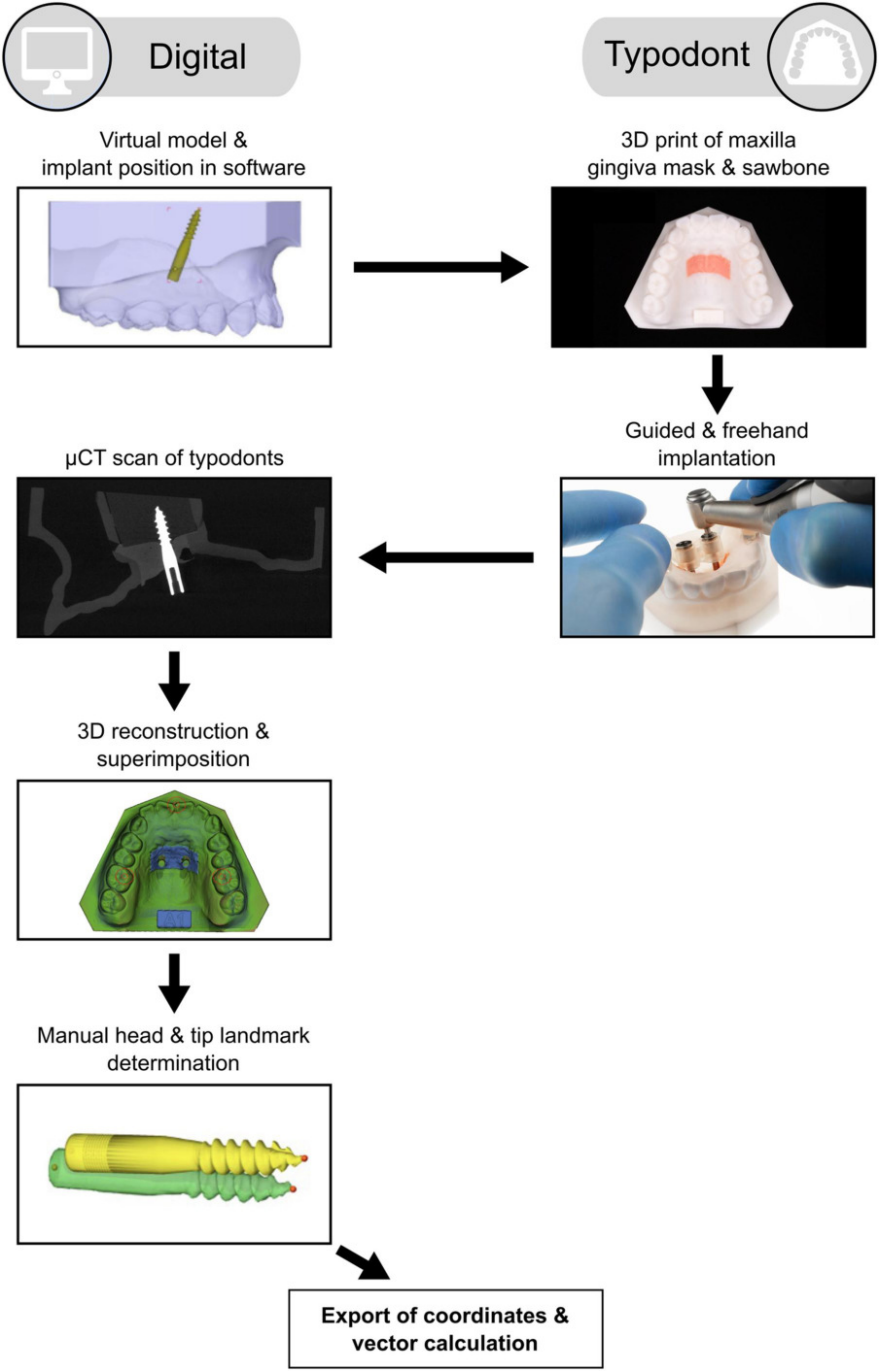
Workflow of the study, including virtual (digital) and hands-on (typodont) steps.

For the present study, the C.R.I.S. guidelines for reporting *in vitro* studies were followed to ensure standardized methodology, transparent reporting, and improved reproducibility of the experimental procedures and outcomes (17). Thirty identical 3D-printed typodonts were created based on a Standard Tessellation Language (STL) file for both experimental groups. Each typodont was embedded with an artificial bone substitute (Solid Foam, 40 PCF, Sawbones, Malmö, Sweden) and covered with a silicone-based material (Gingivafast Rigid, Zhermack GmbH, Marl, Germany) to simulate palatal soft tissue (Figure 1). The study's digital and hands-on workflow is illustrated in Figure 2.

### Placement of temporary anchorage devices

The typodont STL file, obtained with an intraoral scanner (iTero Element 2; Align Technologies, San Jose, CA, USA), was imported into specialized orthodontic software OnyxCeph (Image Instruments, Chemnitz, Germany) for digital TAD planning. Each TAD was virtually positioned parallel and paramedian to the midsagittal plane. The same TAD type was used for all insertions: self-drilling orthodontic mini-implants measuring 14 mm in length and 2.4 mm in diameter (Tiger Dental GmbH, Hörbranz, Austria). No pilot drilling was performed. TADs were inserted directly, either freehand or with the CAD/CAM-milled static guide, according to group allocation.

Before the experiment, both operators were allowed to explore and inspect the 3D digital model of the typodonts and the planned virtual TAD positions. The virtual TAD positions were displayed on a 21-inch monitor and could be interactively viewed and adjusted using a computer mouse. Both clinicians had previous experience with implant placement; the orthodontist was additionally trained in TAD placement. No formal crossover design was applied.

In the freehand group, 30 TADs were placed across 15 typodonts by the orthodontist (S.P.) and the oral surgeon (D.D.) according to random allocation. Randomization between the surgeon and the orthodontist was done using opaque, sealed envelopes. They contained a sheet with the assigned Typodont ID. The IDs were then allocated accordingly. Both clinicians used a prosthodontic screwdriver (iSD900, Nakanishi Inc., Tochigi, Japan) for TAD insertion, operating at a drilling speed of 25 rotations per minute with a maximal torque of 40 Ncm.

Data for the milled guide group were obtained from a previous preclinical study conducted by our research team, which employed a similar experimental design (14). In this group, TADs were placed using static milled guides. Typodonts were produced from the same STL file using the same 3D printer and resin as in the freehand group. TAD insertion was performed with the same calibrated cordless prosthodontic screwdriver, set to 5 rpm and 40 Ncm torque, as used in the freehand group. A hexagonal adapter connected the screwdriver to the TAD, and a metal ring provided a vertical stop. The operator inserted each TAD while applying firm pressure to the guide toward the occlusal surface. Typodonts were positioned base-down on an anti-slip mat under constant laboratory lighting and environmental conditions. All 30 TADs were placed in random order by one experienced orthodontist (A.S.).

### CAD/CAM milled guides

**Figure 3:**
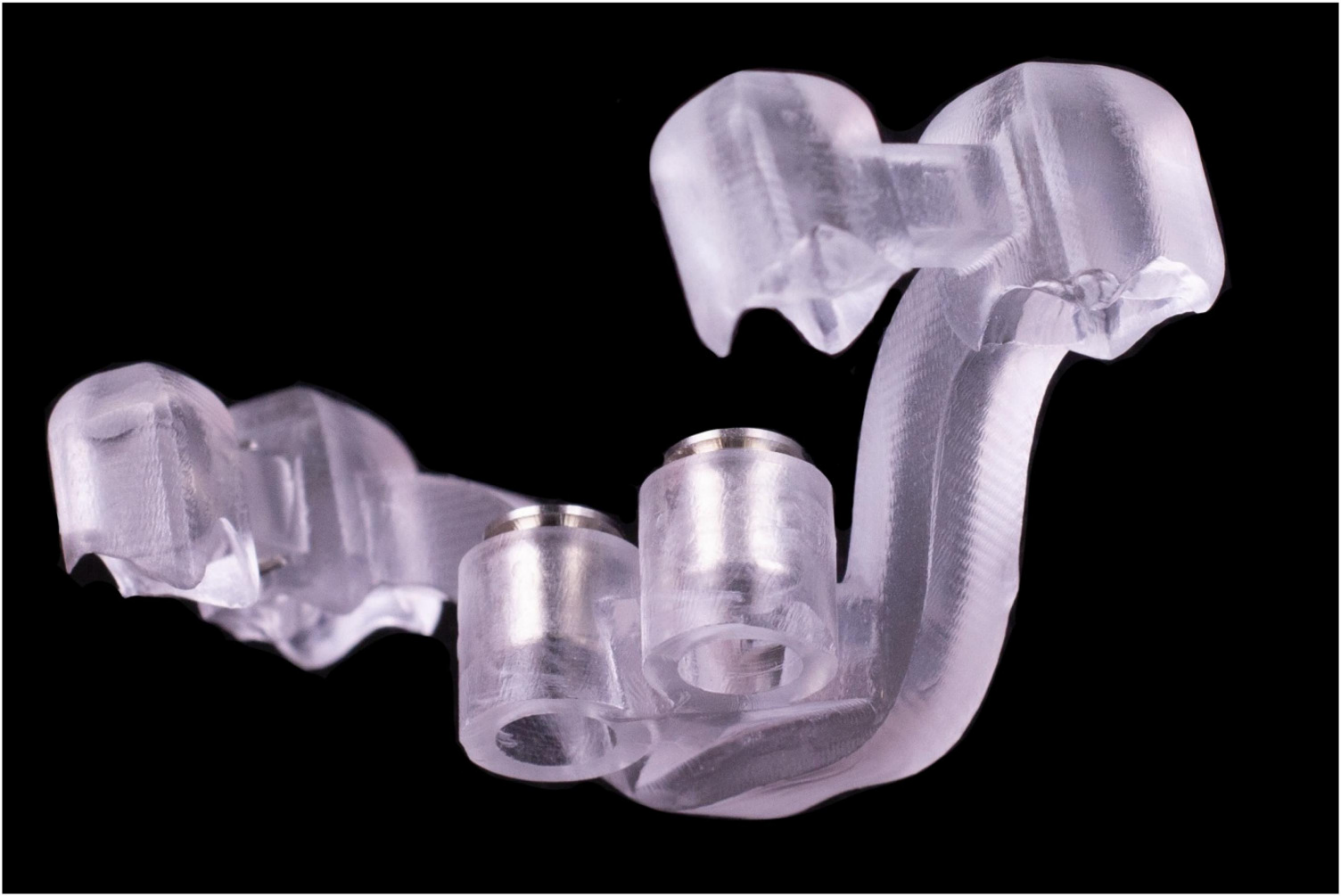
CAD/CAM milled transfer guide.

The guides were designed in the orthodontic software OnyxCeph (Image Instruments, Chemnitz, Germany) by A.S. and milled from transparent polycarbonate material (DD Bio Splint C, Dental Direkt GmbH, Spenge, Germany) using a Programill PM7 milling device (Ivoclar Vivadent AG, Switzerland). The material shows a flexure strength of ≥98 MPa and it is a “Class 1 Medical Device” biocompatible (ISO 20,795–1:2013) and sterilisable by an autoclave up to 121° Celsius (Figure 3).

### TAD deviation measurement

To measure deviations, micro-CT scans of the implanted TADs were overlaid on the corresponding planning models. Each typodont was scanned with following micro-CT settings:

Image reconstruction was performed using XVR-CT, with each scan cropped with voxel resolution set to 50 µm (Table 1). The images were then converted into Taged Image File Format (TIFF) files and later exported as Digital Imaging and Communications in Medicine (DICOM) files through Amira 3D (Thermo Fisher Scientific Inc., Waltham, USA), ensuring compatibility with OnyxCeph software. This step was essential because OnyxCeph requires DICOM files with embedded voxel size metadata. For precise superimposition, grayscale import values were adjusted between 0 and 40.000 to optimize visualization of both, the TADs and typodont structures. The models were then overlaid onto the planning model using the “Register 3D” function within the “Combine 3D” module in OnyxCeph, which combines manual landmark selection with an Iterative Closest Point (ICP) algorithm for greater accuracy.

**Table 1:** Parameters applied for micro CT scans.

Model	Viscom X8060, Viscom AG, Hanover, Germany
Current	320 µA
Voltage	150 kV
Exposition time	1400 ms
Degrees per step	0.25°

The reliability of the superimposition process was assessed by measuring the Hausdorff distances between the corresponding meshes of 15 models, using MeshLab (Institute of Information Science and Technologies “Alessandro Faedo”, Pisa, Italy). Deviation measurements were calculated through vector-based analysis, based on manually identified landmarks (i.e., head and tip of TAD) on both the planned and implanted TADs. The researchers (A.S. and P.C.) marked and recorded the positions of the central point of the TAD head and tip with the “Digitize 3D” module. Both observers were blinded to the group allocation.

Linear and three-dimensional deviations between the actual and planned TADs were expressed as a vector created between the respective heads and apices. Using vector magnitude calculations, the lengths of the three- and two-dimensional (vertical, horizontal, transversal) vectors were determined.

Angular deviations required the construction of a vector from the head to the tip. The scalar product was then formed from the resulting planning vector $\vec{A}$ and the implant vector $\vec{B}$ (Equation 1). This is then divided by the product of the vector lengths, which were calculated as stated above. The inverse cosine function and the subsequent multiplication with 180/*π* allowed for conversion of the value into an angular deviation expressed in degrees.

$$\mathrm{Angular}\;\mathrm{Deviation}=\mathrm{co}s^{-1}\left(\frac{\vec{A}\cdot \vec{B}}{\left|\vec{A}||\vec{B}\right|}\right)\frac{180}{\pi }$$ (1)

### Sample size calculation

A power analysis determined that a clinically significant deviation of 0.14 mm could be detected with a statistical power of 1-*β* = 0.9. Adjustments for multiple testing resulted in a required sample size of *n* = 15 per group. These calculations were based on a standard deviation of 0.3 mm, derived from the interquartile range of mean absolute deviations reported by Mang de la Rosa et al. (18) and served as the base for a previous study comparing milled guides to 3D-printed guides (14).

### Statistical analysis

As an initial step, both intra-observer and inter-observer reliability were evaluated. Intra-class correlation coefficients were calculated to compare the first measurements taken by both investigators, as well as to assess consistency between the first and second measurements of each investigator. Descriptive statistics are provided in both, table and boxplots.

The arithmetic mean of the four measurements per TAD was determined for inferential analysis. Linear mixed models were generated with the fixed effects Group and Quadrant, as well as their interaction. Because multiple measurements were obtained from the same item, observations within items were not independent. To account for this dependency and for baseline differences between items, Items were included as a random intercept. Model assumptions were evaluated through visual inspection of regression plots. The significance calculations based on these models were conducted with the help of a Kenward–Roger approximation at a significance level of 0.05. A Holm–Bonferroni correction was applied to account for multiple comparisons. All calculations were carried out using R version 4.3.3.

## Results

**Figure 4:**
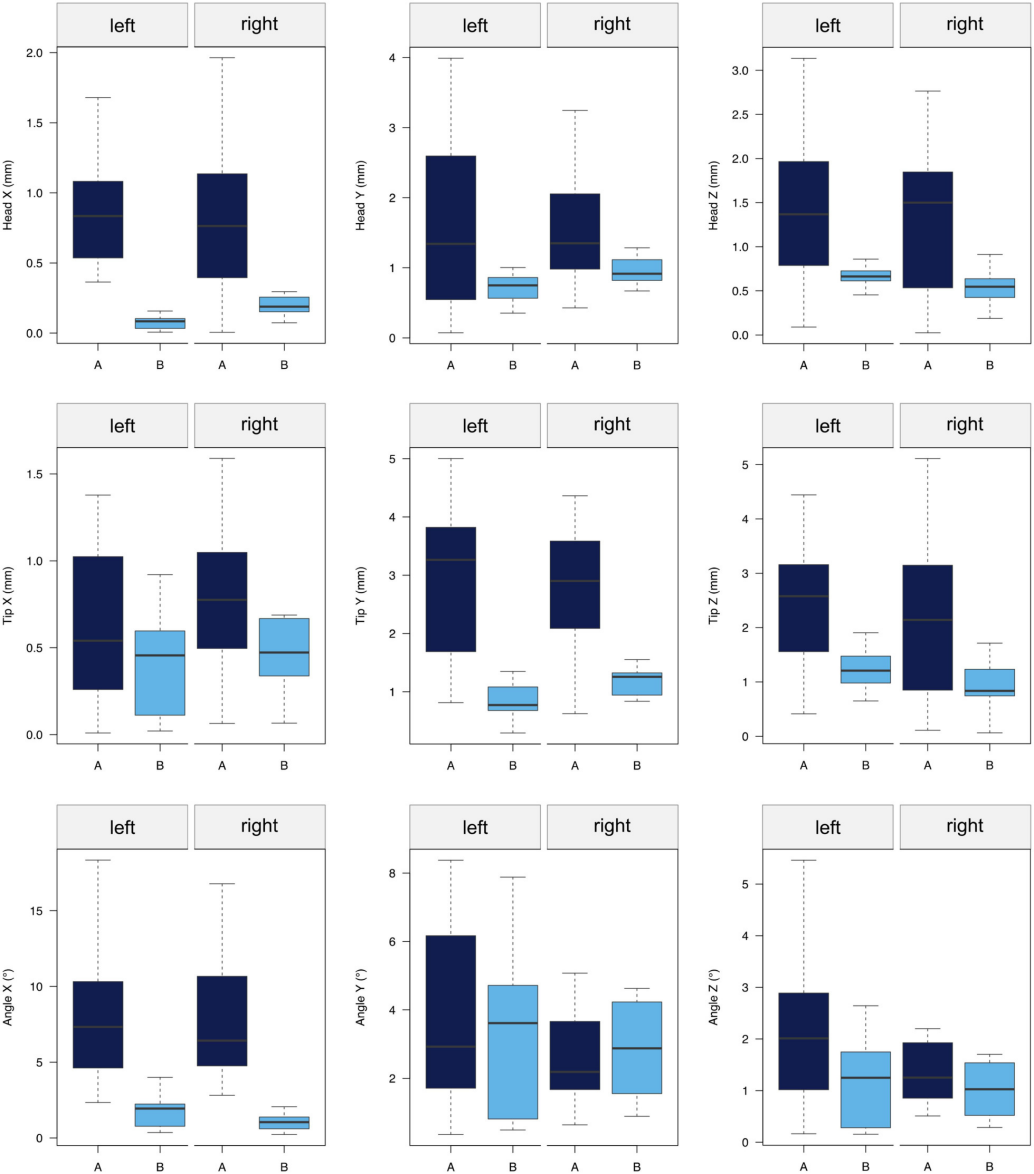
Linear and angular deviations the “freehand” group **(A)** and the “mill” group **(B)** are illustrated in boxplots. Left and right sides of the model are compared.

A total of 60 TADs were successfully placed in 30 models. Deviations between the milled surgical guides and the planning model, as well as those between freehand implantations and the same planning model, were analyzed. Boxplots illustrate the deviations across groups and sides for the head and tip of TADs, as well as for angular deviations (Figure 4).

### Linear, angular and 3D deviations

At the head of the TAD, the deviations (mean ± SD) for the freehand and mill groups ranged from 0.78 ± 0.51 to 1.64 ± 1.03 mm, as well as 0.07 ± 0.05 to 0.95 ± 0.20 mm, respectively. The apex revealed deviations from 0.64 ± 0.47 to 2.95 ± 1.29 mm and 0.40 ± 0.30 to 1.25 ± 0.38 mm, for the freehand and mill groups, respectively. As shown in (Figure 4) the TAD head and tip, linear deviations in X, Y and Z were significantly lower in the mill group (*p* < 0.01 for all except for Tip X, *p* = 0.026 for Tip X).

Angular deviations between planned and actual TAD position ranged from 1.36 ± 0.61° to 7.95 ± 4.55° for the freehand group, and 1.18 ± 0.86° to 3.62 ± 2.86° for the mill group. 3D Angular deviations, as well as angular deviations in X, were significantly lower for the mill group (*p* < 0.01). Angular deviations in Y and Z between the two groups were not statistically significant.

The mean 3D deviations for the head of the TADs were 2.49 mm (left) and 2.41 mm (right) for the freehand group. The mill group showed mean deviations of 0.99 mm (left) as well as 1.13 mm (right). For the tip of the TADs, the freehand group demonstrated mean 3D deviations of 4.04 mm (left) and 3.72 mm (right). Mean 3D deviations for the mill group were 1.64 mm (left) and 1.69 mm (right). Both at the head and the tip, the 3D deviations for the mill group were significantly lower than those measured for the freehand group (*p* < 0.01) (Tables 2 and 3).

**Table 2:** Descriptive data for the measured deviations.

Location	Side	Group	Mean	SD	Min	Q_1	Median	Q_3	Max	*n*
Head X	left	Freehand	0.83	0.36	0.36	0.54	0.83	1.08	1.68	15
		Mill	0.07	0.05	0.01	0.03	0.08	0.10	0.16	15
	right	Freehand	0.78	0.51	0.01	0.40	0.76	1.14	1.96	15
		Mill	0.20	0.10	0.07	0.15	0.19	0.26	0.44	15
	All	Freehand	0.81	0.44	0.01	0.49	0.78	1.10	1.96	30
		Mill	0.14	0.10	0.01	0.08	0.12	0.18	0.44	30
Head Y	left	Freehand	1.61	1.30	0.07	0.55	1.34	2.59	3.99	15
		Mill	0.71	0.20	0.35	0.57	0.75	0.86	1.00	15
	right	Freehand	1.64	1.03	0.43	0.98	1.35	2.05	3.85	15
		Mill	0.95	0.20	0.67	0.82	0.92	1.11	1.29	15
	All	Freehand	1.62	1.15	0.07	0.72	1.35	2.18	3.99	30
		Mill	0.83	0.23	0.35	0.72	0.84	0.96	1.29	30
Head Z	left	Freehand	1.39	0.88	0.09	0.79	1.37	1.97	3.14	15
		Mill	0.67	0.17	0.38	0.61	0.66	0.73	1.06	15
	right	Freehand	1.27	0.84	0.03	0.53	1.50	1.85	2.76	15
		Mill	0.55	0.19	0.19	0.42	0.55	0.64	0.91	15
	All	Freehand	1.33	0.85	0.03	0.64	1.41	1.90	3.14	30
		Mill	0.61	0.19	0.19	0.47	0.63	0.70	1.06	30
Tip X	left	Freehand	0.64	0.47	0.01	0.26	0.54	1.02	1.38	15
		Mill	0.40	0.30	0.02	0.11	0.46	0.60	0.92	15
	right	Freehand	0.79	0.44	0.06	0.50	0.78	1.05	1.59	15
		Mill	0.56	0.37	0.07	0.34	0.47	0.67	1.47	15
	All	Freehand	0.72	0.45	0.01	0.43	0.68	1.04	1.59	30
		Mill	0.48	0.34	0.02	0.24	0.46	0.64	1.47	30
Tip Y	left	Freehand	2.95	1.29	0.81	1.69	3.26	3.82	5.00	15
		Mill	0.86	0.29	0.29	0.68	0.77	1.08	1.35	15
	right	Freehand	2.75	1.10	0.63	2.09	2.90	3.59	4.36	15
		Mill	1.18	0.24	0.84	0.94	1.26	1.32	1.55	15
	All	Freehand	2.85	1.18	0.63	1.84	3.12	3.63	5.00	30
		Mill	1.02	0.31	0.29	0.79	1.02	1.28	1.55	30
Tip Z	left	Freehand	2.54	1.47	0.41	1.56	2.58	3.16	5.80	15
		Mill	1.25	0.38	0.65	0.98	1.21	1.47	1.91	15
	right	Freehand	2.18	1.60	0.11	0.85	2.14	3.15	5.11	15
		Mill	0.93	0.46	0.06	0.75	0.84	1.23	1.71	15
	All	Freehand	2.36	1.52	0.11	0.92	2.50	3.17	5.80	30
		Mill	1.09	0.44	0.06	0.80	1.13	1.38	1.91	30
Angle X	left	Freehand	7.89	4.31	2.34	4.62	7.33	10.32	18.34	15
		Mill	1.66	1.00	0.36	0.77	1.94	2.24	4.01	15
	right	Freehand	7.95	4.55	2.82	4.76	6.43	10.67	16.78	15
		Mill	1.18	0.86	0.22	0.60	1.04	1.38	3.06	15
	All	Freehand	7.92	4.35	2.34	4.49	6.93	10.76	18.34	30
		Mill	1.42	0.95	0.22	0.68	1.09	2.13	4.01	30
Angle Y	left	Freehand	4.02	2.78	0.36	1.71	2.93	6.17	8.38	15
		Mill	3.46	2.46	0.49	0.81	3.61	4.72	7.88	15
	right	Freehand	2.61	1.28	0.64	1.67	2.19	3.66	5.08	15
		Mill	3.62	2.86	0.89	1.55	2.88	4.23	10.04	15
	All	Freehand	3.32	2.24	0.36	1.64	2.82	4.63	8.38	30
		Mill	3.54	2.62	0.49	1.05	3.41	4.60	10.04	30
Angle Z	left	Freehand	2.34	1.87	0.16	1.02	2.01	2.89	6.63	15
		Mill	1.20	0.85	0.16	0.28	1.25	1.75	2.64	15
	right	Freehand	1.36	0.61	0.51	0.85	1.25	1.93	2.20	15
		Mill	1.29	1.03	0.29	0.52	1.02	1.54	3.58	15
	All	Freehand	1.85	1.45	0.16	0.83	1.47	2.16	6.63	30
		Mill	1.24	0.93	0.16	0.39	1.21	1.70	3.58	30

**Table 3:** Results in linear, angular and 3D deviations including the *p*-values from linear mixed model.

Location	Milled guide (average ± SD)	Freehand (average ± SD)	*p*-value
Linear deviations (mm)			
Head deviation in X	0.14 ± 0.1	0.81 ± 0.44	***p*** **<** **0.001**
Head deviation in Y	0.83 ± 0.23	1.62 ± 1.15	***p*** **<** **0.001**
Head deviation in Z	0.61 ± 0.19	1.33 ± 0.85	***p*** **<** **0.001**
Tip deviation in X	0.48 ± 0.34	0.72 ± 0.45	***p*** **=** **0.026**
Tip deviation in Y	1.02 ± 0.31	2.85 ± 1.18	***p*** **<** **0.001**
Tip deviation in Z	1.09 ± 0.44	2.35 ± 1.52	***p*** **<** **0.001**
Angular deviations (°)			
Angular deviation in X	1.42 ± 0.95	7.92 ± 4.35	***p*** **<** **0.001**
Angular deviation in Y	3.54 ± 2.62	3.32 ± 2.24	*p* = 0.725
Angular deviation in Z	1.24 ± 0.93	1.85 ± 1.45	*p* = 0.059
3D deviations			
Head (mm)	1.06 ± 0.23	2.45 ± 1.12	***p*** **<** **0.001**
Tip (mm)	1.66 ± 0.29	3.88 ± 1.73	***p*** **<** **0.001**
Angular (°)	2.07 ± 0.88	8.77 ± 4.86	***p*** **<** **0.001**

Bold *p*-values indicate statistically significant deviations.

### Differences between orthodontist and oral surgeon

Significant differences were found between the screws placed by either an oral surgeon or an orthodontic specialist. The TADs placed by orthodontist displayed significantly higher 3D- and linear deviations in X and Y at the head (*p* < 0.01). At the tip, TADs placed by orthodontist showed significantly higher 3D- and linear deviations in Y and Z (*p* < 0.01). No significant differences between the two operators were detected regarding angular deviations (*p* = 0.7635). The comparison between operators should be considered exploratory in nature. Due to the small subgroup sizes (*n* = 7 and *n* = 8), this analysis was not powered for definitive conclusions and should be interpreted accordingly. A *post-hoc* analysis revealed a statistical power of 1-*β* = 0.45.

### Interclass correlation

All measurement points on the head and the tip where set twice by both raters (A.S. and P.C.). The intraclass correlation coefficient (ICC) for the first and second runs of the observers showed excellent correlation (Table 4). Also, the ICC comparing the measurements between the investigators ranged from good (0.88) to excellent (0.99) as shown in Table 5.

**Table 4:** ICC for intra-rater agreement.

Location	ICC	Lower bound	Upper bound
Head	0.999	0.998	0.999
Tip	0.999	0.999	0.999
Angle	0.99	0.987	0.993

**Table 5:** ICC for inter-rater agreement.

Location	ICC	Lower bound	Upper bound
Head	0.998	0.997	0.999
Tip	0.999	0.998	0.999
Angle	0.869	0.835	0.89

## Discussion

The present study directly compared the transfer accuracy of orthodontic miniscrews placed using CAD/CAM-milled surgical guides versus freehand insertion. Guided placement yielded significantly lower deviations across all spatial dimensions, indicating superior precision.

These findings reject the null hypothesis and support the routine use of CAD/CAM-milled guides, particularly in situations requiring high positional accuracy such as simultaneous placement of TADs and associated orthodontic appliances.

In particular, 3D deviations at both the TAD head and tip were more than twice as large in the freehand group. Head deviations measured approximately 2.5 mm freehand compared to around 1 mm with guided placement, while tip deviations reached 4 mm freehand compared with 1.7 mm in the guided group. Moreover, standard deviations were consistently smaller in the guided group, demonstrating not only greater accuracy but also improved reproducibility. In addition, although the oral surgeon achieved higher accuracy with freehand insertion than the orthodontist, both performances remained significantly inferior to guided placement. These findings highlight the value of CAD/CAM-milled guides in enhancing placement predictability.

In orthodontics, the anterior palate, particularly the area posterior to the third palatal rugae, has been identified as one of the most favorable sites for TAD placement (19–22). Wilmes et al. (2016) introduced the term “T-Zone” to describe this region, recommending paramedian placement for sagittal tooth movement and maxillary expansion, and median placement for vertical control and palatal expansion in cases with displaced upper canines (23). This site is characterized by thin soft tissue and minimal injury risk to roots, nerves, or blood vessels (24). Although insertion guides can enhance placement accuracy, Iodice et al. (2022) suggest that freehand placement remains a safe and viable option in many cases (25). Still, while freehand insertion is more commonly used, drilling templates offer clear advantages in complex cases, such as limited bone availability, palatally displaced teeth, or cleft palate, and may be especially beneficial for less experienced clinicians (26). Moreover, a bicortical TAD anchorage, beneficial for biomechanical effectiveness, is also facilitated through guided TAD placement (5). Greater precision also implies lower risk of root damage, cortical perforation, or failure of immediate loading, particularly in anatomically sensitive areas or when bicortical engagement is required. In view of the relatively high failure rates reported for orthodontic miniscrews, averaging 13%–16% and reaching up to 30% in some studies, which are often linked to inaccurate insertion such as root contact, the present study provides a timely and relevant analysis of placement accuracy (27–29).

Notably, most prior studies have focused on additive (printed) manufacturing techniques for guide fabrication (16, 18, 26, 30, 31). In contrast, our study is among the first to investigate milled (subtractive) guides, highlighting comparable or even superior outcomes, with potential differences in material stability and design precision. The findings from the present research are consistent with previous studies reporting superior accuracy with guided TAD placement. Qiu et al. (2012) demonstrated that photopolymerized resin guides significantly reduced linear and angular deviations compared to freehand placement, with no observed root damage (32). Suzuki and Suzuki (2008) reported head and tip deviations of 0.6 ± 0.5 mm and 2.0 ± 0.4 mm, respectively, using 3D guides, compared to 3.6 ± 1.7 mm and 10.5 ± 3.5 mm in the freehand group. Angular deviations were also substantially lower with guides: 1.8 ± 0.9° compared to 21.2 ± 2.9° for freehand placement (33). The present results follow a comparable trend, further validating the benefit of guided placement. However, recent evidence indicates that full-arch surgical guide designs demonstrate significantly higher accuracy than the skeletonized design employed in this study across most spatial dimensions, though both approaches remain superior to freehand placement in reducing angular and linear deviations (30, 34). A clinical study by Möhlhenrich et al. stated that the fabrication method may influence accuracy, as pressure-molded guides showed significantly lower angular deviations compared to 3D-printed guides (3.61° vs. 5.77°, *p* = 0.006), though both remained within clinically acceptable ranges (35). While the skeletonized design offers advantages in patient comfort and intraoperative visibility, clinicians should recognize this accuracy trade off when maximum precision is required (30).

The fabrication method (Milling vs. 3D-printing) appears to have minimal impact on accuracy, as a previous study by our group demonstrated that while CAD/CAM milled guides showed numerically higher accuracy with lower standard deviations compared to 3D-printed guides, these differences were not statistically significant, and clinical studies are needed to confirm these preclinical findings (14).

When comparing the freehand group deviations between the TADs placed by either an oral surgeon or orthodontist, we measured significant differences in almost all distance dimensions. TADs placed by the oral surgeon demonstrated significantly greater accuracy than those placed by the orthodontist, with smaller 3D and X and Y deviations at the head and reduced 3D, Y, and Z deviations at the tip. This raises the question as to which factors contributed to the higher accuracy achieved by the oral surgeon in several categories. In addition, it is worth investigating to what extent the oral surgeon's more frequent practice of guided or freehand insertion of dental implants plays a role for orthodontic miniscrew insertions. Clinician-dependent factors and differences between various experience levels require further research. Likewise, it is also of interest whether the clinician's experience or area of expertise affects the transfer accuracy in guided placement of miniscrews. A comparable study involving ten experienced surgeons placing implants in plastic models also found that freehand placement was significantly less accurate, reinforcing our findings (36).

To assess bone availability for TAD placement near the upper first premolars, lateral cephalograms can be used, however, two-dimensional imaging has limitations, including distortion and magnification errors (31, 37, 38). CBCT by contrast, provides a more accurate assessment of interradicular space, thus improving placement predictability (39). For the virtual planning of the TAD positions, a standard protocol includes CBCT scan, however, for the evaluation of the final TAD position, a high-resolution micro-CT imaging was used. Micro-CT offers markedly superior spatial resolution and reduced image-artifact influence compared to CBCT, making it the preferred modality for precise assessment of final implant positioning. While CBCT systems typically achieve voxel sizes in the range of 75–400 µm, micro-CT systems routinely reach voxel resolutions of 5–50 µm, enabling more accurate 3D visualization of implant position (40, 41). Because of these advantages such as higher resolution and more accurate quantification of linear and angular deviations, we selected micro-CT for our accuracy study.

The *in vitro* study design, while enabling controlled conditions and the use of high-resolution micro-CT imaging, does not fully replicate the clinical environment. Important biological and procedural variables intrinsic to *in vivo* implant placement, such as soft-tissue thickness, variability in bone quality and density, patient movement, saliva, and often bleeding, were absent in this study and could influence surgical accessibility, drill stability, and ultimately TAD placement accuracy. Likewise, limited mouth opening, neighboring teeth, and patient-specific anatomical variations were not represented, potentially overestimating the precision achievable in clinical practice. The artificial bone material used offers standardized morphology and homogeneity, but it cannot accurately mimic the anisotropic structure, cortical–trabecular relationships, or varying mineral densities of human bone, which may alter drilling behavior and primary stability.

The data support improved accuracy under controlled preclinical conditions, but they do not establish clinical superiority, cost-effectiveness, or the need for guided placement in routine TAD insertion. While statistically significant differences in angular and positional deviation were observed *in vitro*, the clinical relevance of these differences remains uncertain. No evidence was provided regarding whether the improvements translate to measurable differences in treatment outcomes, complication rates, or TAD survival. The study does not address the learning curve, fabrication time, or economic feasibility of guided protocols. Clinicians should weigh potential precision gains against practical considerations, recognizing that freehand placement remains well established and effective for most orthodontic applications.

A potential limitation is operator-related variability in the freehand group, as TADs were placed by both an orthodontist and an oral surgeon. Although typodonts were randomly allocated and both operators were familiarized with the protocol and virtual TAD positions, no formal calibration or crossover design was performed. Therefore, differences in clinical background and TAD-specific training may have influenced placement accuracy. The comparison between the orthodontist and the oral surgeon should also be interpreted carefully. If operator-related accuracy was an important question, both operators should ideally have placed TADs under both conditions, freehand and guided, preferably in a randomized crossover or factorial design. With the current design, the data do not allow robust conclusions about whether guided placement compensates for operator experience or whether one professional group is generally more accurate.

In addition, the guide placement and TAD insertion were performed under ideal visibility and access conditions, without the limitations imposed by clinical ergonomics, soft-tissue management, or patient discomfort. Clinical success also depends on anatomical planning, operator experience, soft-tissue conditions, patient-related factors, guide seating, access, visibility, and biomechanical requirements, which were not addressed in this study. These factors collectively suggest that, although micro-CT measurements provide a highly accurate assessment of transfer precision *in vitro*, the true clinical performance of milled guides may be influenced by additional biological and operative variables. Therefore, clinical studies are needed to confirm the transferability of these findings to real-world conditions.

## Conclusion

Within the limitations of this *in vitro* study, CAD/CAM-milled surgical guides showed higher transfer accuracy and lower variability than freehand TAD placement. Although operator experience in dental implantology improved freehand accuracy, guided insertion remained more precise. Future clinical studies should validate these findings and further assess the influence of operator experience in dental implantology.

## Data Availability

The raw data supporting the conclusions of this article will be made available by the authors after conformation by the Medical University of Vienna.
